# Neuroprotective effects of Pycnogenol on nerve regeneration and functional recovery after sciatic nerve crush injury in rodents

**DOI:** 10.1038/s41598-025-21975-7

**Published:** 2025-10-30

**Authors:** Bhavani R. Nayak, Nanda Acharya, Dhiren Punja, Akash Tomar, Ashwath M. Acharya, Rohini Punja, Nihaal Maripini, Rathi Bishakha Jayprakash

**Affiliations:** 1 Department of Physiology , Kasturba Medical College, Manipal, Manipal Academy of Higher Education , Manipal, India; 2 Department of Hand surgery , Kasturba Medical College, Manipal, Manipal Academy of Higher Education, Manipal, India; 3 Department of Anatomy, Kasturba Medical College, Manipal, Manipal Academy of Higher Education, Manipal, India; 4 Department of Biochemistry , Kasturba Medical College, Manipal, Manipal Academy of Higher Education, Manipal, India; 5 Department of Pharmacology , Kasturba Medical College, Manipal, Manipal Academy of Higher Education, Manipal, India

**Keywords:** Pycnogenol, Sciatic nerve regeneration, Peripheral nerve injury, Neuroprotection, Sprague Dawley rats, Sciatic nerve crush injury, Neuroscience, Physiology, Anatomy, Neurology

## Abstract

Pycnogenol, a standardized French maritime pine bark extract, is known for its antioxidant, anti-inflammatory, and neuroprotective properties. This study evaluated its therapeutic potential in sciatic nerve regeneration following crush injury in female Sprague Dawley rats. Twenty-one rats were assigned to Sham, Control, and Pycnogenol (100 mg/kg/day) groups. After standardized nerve injury, Pycnogenol was administered for 28 days. Functional recovery was assessed using the Sciatic Functional Index (SFI), pinprick, and cold allodynia tests. Histopathology, muscle weight, and ELISA for nerve growth factor (NGF) were evaluated post-euthanasia. By Day 14, the Pycnogenol group showed significantly better SFI scores (83.60 ± 2.26 vs. 89.81 ± 2.42, *p* < 0.001), with continued improvement through Day 28 (49.42 ± 3.0 vs. 62.95 ± 2.93, *p* < 0.001). Histological analysis revealed enhanced muscle regeneration, increased fiber area, and improved myelination. ELISA confirmed significantly elevated NGF levels, supporting Pycnogenol’s neuroprotective role. These findings highlight its potential in peripheral nerve injury treatment. Further research is needed to confirm efficacy in humans, explore molecular mechanisms, and compare it with existing neuro-regenerative therapies. Pycnogenol may serve as a promising agent in neurology and regenerative medicine.

**Supplementary Information:**

The online version contains supplementary material available at 10.1038/s41598-025-21975-7.

## Introduction

Peripheral nerve injury (PNI), commonly caused by crushing, stretching, penetrating trauma, or roadside accidents, disrupts sensory and motor nerve functions. This leads to restricted movement and, in severe cases, irreversible physical disability. These injuries trigger Wallerian degeneration, a process where the distal axon and myelin sheath degenerate following axonal discontinuity, followed by a slow regenerative phase dependent on Schwann cell activity and neurotrophic support^1^. Wallerian degeneration begins by 24–48 h post-injury and is characterized by calcium influx, breakdown of the axonal cytoskeleton and activation of calpains. Schwann cells undergo phenotypic transformation, upregulating neurotrophic factors and forming Bands of Büngner to guide axonal regrowth. However, delayed repair, fibrotic scarring or prolonged denervation can significantly hamper this regenerative process^1^. The regenerative capacity of peripheral nerves is influenced by multiple factors including age, injury severity, gap length, and the inflammatory microenvironment. While spontaneous regeneration can occur, functional recovery is often incomplete, particularly in severe injuries or when repair is delayed beyond the optimal time window^2^. Damage to Schwann cells and axonal structures compromises neural integrity, making functional recovery a slow and challenging process^2,3^. Traumatic PNIs often result in significant and long-lasting functional impairments, including sensory deficits, muscle weakness, and chronic pain^4^. Various pharmacological interventions have been employed to accelerate and enhance sensory and motor function recovery, but they generally show limited success in promoting nerve regeneration^5,6^.

Plants are abundant sources of bioactive phytochemicals and have long been explored for their potential in drug discovery. Due to their natural safety profile and diverse pharmacological effects, plant-derived compounds have gained prominence as potential alternatives to synthetic drugs^7–9^. Natural products like Curcumin and Quercetin^10,11^ have demonstrated therapeutic potential against various neurodegenerative diseases, including Alzheimer’s disease, Parkinson’s disease, and Huntington’s disease^12–14^. These compounds often possess antioxidant and anti-inflammatory properties and can modulate cellular signaling pathways involved in neuronal survival, axonal growth, and regeneration^13–15^. Among these, Pycnogenol, a patented extract from French maritime pine bark (*Pinus pinaster* subsp. *atlantica*, Pinaceae), has demonstrated significant pharmacological potential. Manufactured by Horphag Research (Geneva, Switzerland), Pycnogenol is standardized to contain 70 ± 5% procyanidins (*Pycnogenol - American Botanical Council*, 2019)^16,17^. Studies conducted have shown that Pycnogenol exhibits better bioavailability when administered orally, with its polyphenolic constituents being efficiently absorbed and metabolized, leading to sustained plasma levels^18^. Preclinical and clinical studies confirm its excellent safety profile, showing no significant toxicity in animal models even at high doses of up to 1000 mg/kg/day in rodents, and well-tolerated effects in human trials. Its long-term consumption has been deemed safe, with no major adverse effects reported in both experimental and clinical settings^19^. Studies conducted in vitro, in animal models, and in humans have shown that it possesses potent antioxidant^20^ and anti-inflammatory activities^21^, improves endothelial function^22^, suppresses platelet aggregation, and lowers blood glucose levels by inhibiting α-glucosidase activity^23^. It has been found beneficial in managing diabetes-related complications, neuropathy, cardiomyopathy, and liver damage, wound healing, improved skin hydration and elasticity, and protection against sunburn through the modulation of gene expression, mast cell-mediated responses, protects against nephrotoxicity, exhibits anti-cancer and bone-preserving effects^24^, and enhances reproductive health by improving sperm morphology and function^25^.

Pycnogenol has demonstrated neuroprotective effects across multiple neurological conditions. In ADHD clinical trials, Pycnogenol improved attention and hyperactivity scores while normalizing oxidative markers^26^. Animal studies in Alzheimer’s disease models showed decelerated plaque development and enhanced spatial memory^27^. Following traumatic brain injury, Pycnogenol reduced oxidative stress and neuroinflammation while preserving synaptic protein levels^28,29^. In Parkinson’s disease models, it reduced dopaminergic neuron loss and improved motor symptoms through inhibition of inflammatory pathways^30,31^. Clinical studies in healthy adults consistently demonstrated improvements in cognitive function and reduced oxidative stress markers^32^.

Given its diverse pharmacological properties, Pycnogenol presents itself as a promising candidate for the treatment and management of various diseases, including neurodegenerative disorders and neural injuries^26–32^. However, its potential role in promoting peripheral nerve regeneration remains unclear. Therefore, this study aims to investigate the therapeutic effects of Pycnogenol on sciatic nerve regeneration in inbred female Sprague Dawley rats.

## Results

### Sciatic Functional Index (SFI) score

**Fig. 1:**
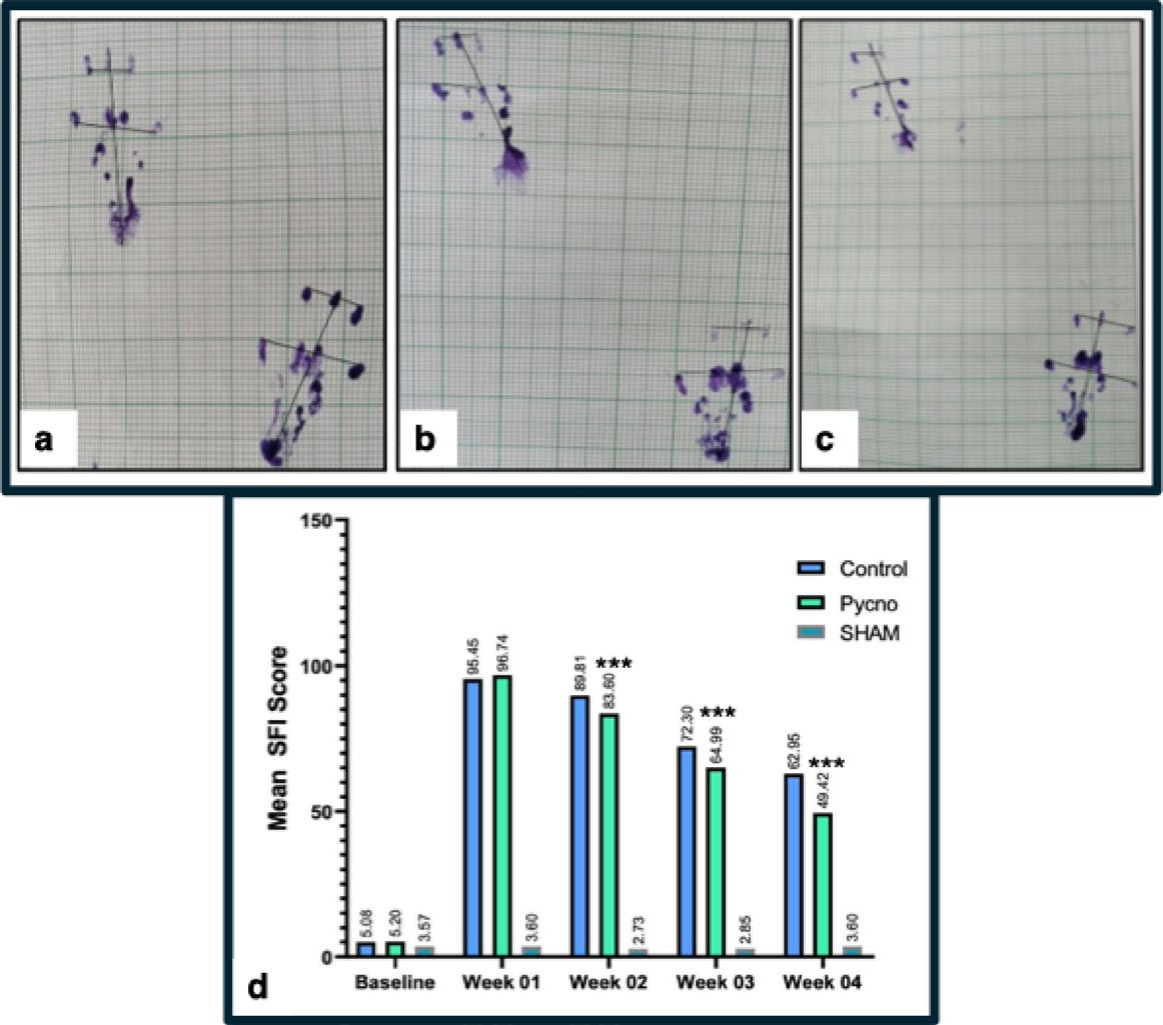
Sciatic function index (SFI) among the three experimental groups. Representative paw prints obtained on Day 28 post-injury are shown in panels (**a**–**c**). Panel (**a**) corresponds to the Sham group, (**b**) to the Control group, and (**c**) to the Pycnogenol-treated group. The paralyzed left hind paw is compared with the intact right paw. Notably, the Pycnogenol group (**c**) exhibits marked improvement in paw placement compared to the Control group (**b**), evidenced by an increased toe spread (TS) and reduced paw length (PL), indicating enhanced functional recovery.(d)Bar graph showing the temporal progression of Sciatic Functional Index (SFI) scores following nerve crush injury(*n* = 7 per group). Two-way repeated measures ANOVA with Tukey’s post-hoc test. ***p < 0.001 vs. respective Control group.

At baseline, there was no significant difference between the Control (5.08 ± 2.33) and Pycnogenol (5.20 ± 2.78) groups (*p* = 0.935), indicating comparable starting conditions. By Day 7, both groups showed increased SFI scores, with no significant difference (*p* = 0.325). By Day 14, the Pycnogenol group exhibited significant improvement (83.60 ± 2.26 vs. 89.81 ± 2.42, ***p < 0.001). This trend continued to Day 28, with the Pycnogenol group showing a score of 49.42 ± 3.0 compared to 62.95 ± 2.93 in the Control group (***p < 0.001).These results are illustrated in Fig. 1 and supplementary Table 1, which integrates paw print images and SFI trends over time.

### Pin Prick test

**Fig. 2:**
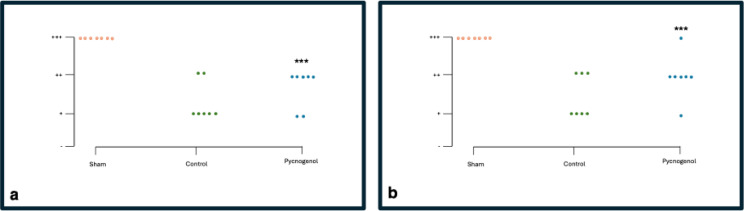
Pin prick response represented as dot graph. (**a**) Pin prick response graph of week 3; The sham group showed uniformly normal responses (+++), indicating preserved sensory integrity. In contrast, the Control group predominantly exhibited mild to moderate responses, suggesting delayed nerve recovery. The Pycnogenol group showed better responses than Control, with a shift toward moderate grades (++). A chi-square test showed a significant difference among groups (χ² = 24.9, df = 4, *p* < 0.001), supported by Fisher’s exact test (***p < 0.001), indicating that treatment significantly influenced sensory recovery.(**b**) Pin prick response graph of week 4.The Sham group maintained consistent (+++) normal responses. The Control group showed a spread across mild (+), moderate (++), and normal (+++) responses, reflecting incomplete recovery. However, the Pycnogenol group exhibited notable improvement from week 3, with a majority showing moderate (++) and one regaining back the normal (+++) response. Statistical analysis (χ² = 20.7, df = 4,*p* < 0.001; Fisher’s exact test ***p < 0.001) confirmed a significant difference in outcomes among the groups, suggesting enhanced sensory regeneration with Pycnogenol treatment.

At baseline, all rats across both groups displayed a normal (+++) response to pinprick stimulation. By Day 21, five rats in the Control group regressed to mild (+), while two shifted to moderate response(++). In the Pycnogenol group, two rats regressed to mild response (+), and five shifted to moderate response (++). By Day 28, the Control group showed four rats in mild (+) and three in moderate response(++). In contrast, the Pycnogenol group demonstrated better sensory recovery, with one rat in mild (+), five in moderate(++), and one accomplishing the normal (+++) response (*p < 0.05 vs. Control, Chi-square test). These findings suggest that Pycnogenol may contribute to an improved sensory response after nerve injury as shown in Fig. 2.

### Cold allodynia test

**Fig. 3:**
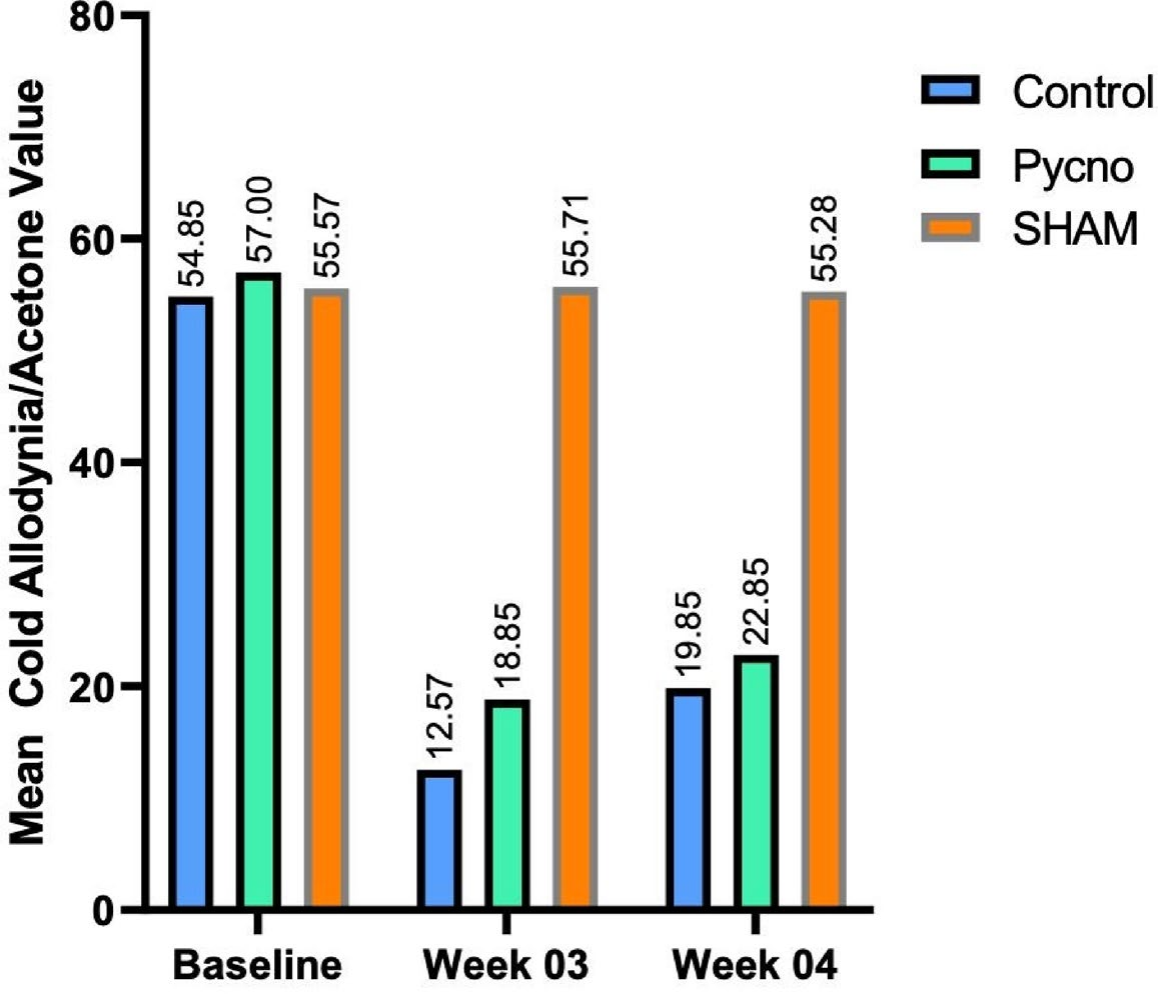
Comparison of mean cold allodynia/acetone value among the study groups at baseline, at weeks 3 and 4 following sciatic nerve crush injury. Cold allodynia responses (Sham: 53.76 ± 3.89, Control: 19.85 ± 1.06, Pycnogenol: 22.85 ± 0.89 on Day 28, ***p < 0.001 vs. Control, one-way ANOVA with Tukey’s post hoc test. The Pycnogenol group shows enhanced sensory recovery.

At baseline, there was no significant difference between the Control (54.85 ± 3.13) and Pycnogenol (57.0 ± 2.16) groups (*p* = 0.162), confirming similar levels of cold allodynia before treatment. By Day 21, the Control group exhibited a mean value of 12.57 ± 1.71, while the Pycnogenol group had a significantly higher value of 18.85 ± 1.86 (***p < 0.001), suggesting enhanced recovery in cold sensitivity. On Day 28, the Control group showed a mean value of 19.85 ± 1.06, while the Pycnogenol group showed better improvement at 22.85 ± 0.89 (***p < 0.001). These results indicate that Pycnogenol treatment effectively alleviates cold allodynia, particularly from Day 21 onwards. (Fig. 3,supplementary Table 2)

### Wet muscle weight

**Fig. 4:**
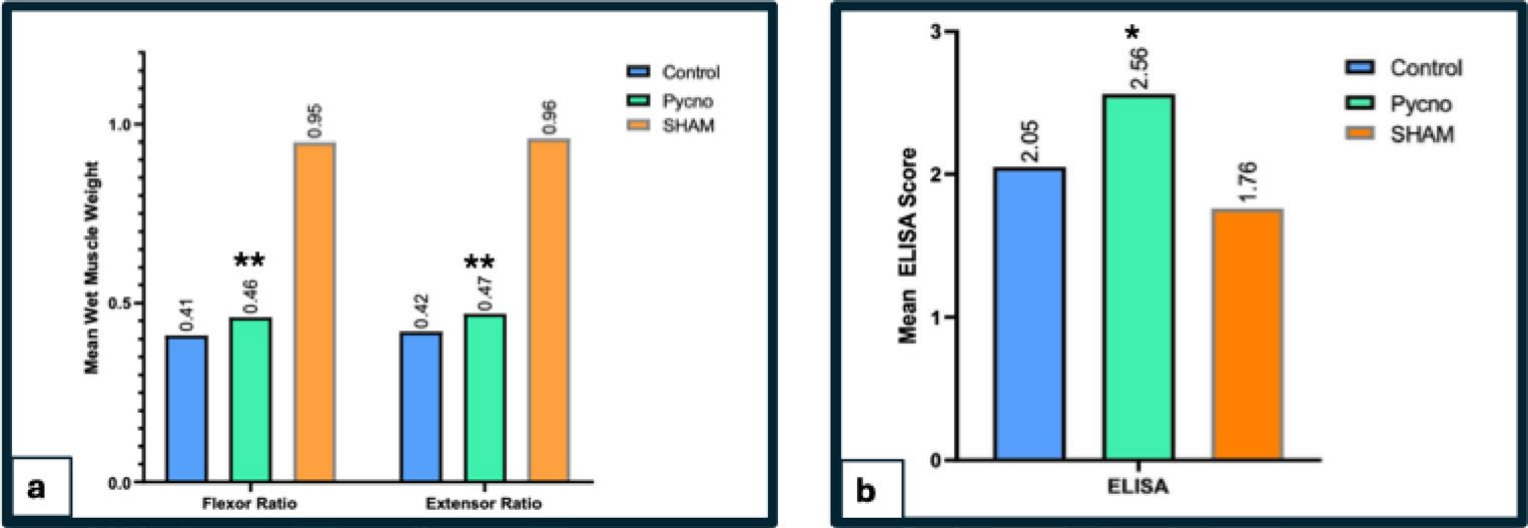
(**a**) Bar graph showing the distribution of wet muscle weight loss percentage for Sham, Control, and Pycnogenol groups (*n* = 7 per group). Flexor ratio (Sham: 0.95 ± 0.01, Control: 0.41 ± 0.021, Pycnogenol: 0.46 ± 0.03),**p = 0.006 and Extensor ratio(Sham: 0.96 ± 0.03, Control: 0.42 ± 0.01, Pycnogenol: 0.47 ± 0.03 **p = 0.008 vs. Control, one-way ANOVA with Tukey’s post hoc test The Pycnogenol group shows significantly higher ratios vs. Control. (**b**) Bar graph representing mean ELISA β-NGF(pg/mg) levels across Sham(1.76 ± 0.21), Control(2.05 ± 0.15), and Pycnogenol-treated groups(2.56 ± 0.43) (*n* = 7 per group. The Pycnogenol group demonstrated a statistically significant increase in β-NGF levels compared to the Control group (*p = 0.013), as determined by one-way ANOVA followed by Tukey’s post hoc test.

Analysis of muscle weight by formula *(Muscle wet weight − loss (%) = (1 − Wet weight of muscle on the injured side/Wet weight of muscle on the uninjured side) × 100)*. Analysis of muscle weight loss percentage (supplementary Table 3, Fig. 4a) showed that the flexor muscle weight ratio was 0.41 ± 0.021 in the Control group and significantly higher at 0.46 ± 0.03 in the Pycnogenol group (**p = 0.006). The extensor muscle weight ratio was 0.42 ± 0.01 in the Control group and 0.47 ± 0.03 in the Pycnogenol group (**p = 0.008), indicating that Pycnogenol supplementation mitigates muscle atrophy post-injury.

### Enzyme-linked immunosorbent assay (ELISA) of β-nerve growth factor (NGF)

Biochemical analysis of β-NGF levels revealed a significant increase in the Pycnogenol-treated group. The Control group exhibited a mean β-NGF level of 2.05 ± 0.15 pg/mg, which increased significantly to 2.56 ± 0.43 pg/mg in the Pycnogenol group (*p = 0.013). These results suggest that Pycnogenol enhances neurotrophic factor expression, which may contribute to nerve regeneration. (supplementary Table 4, Fig. 4b)

### Muscle histology

**Fig. 5:**
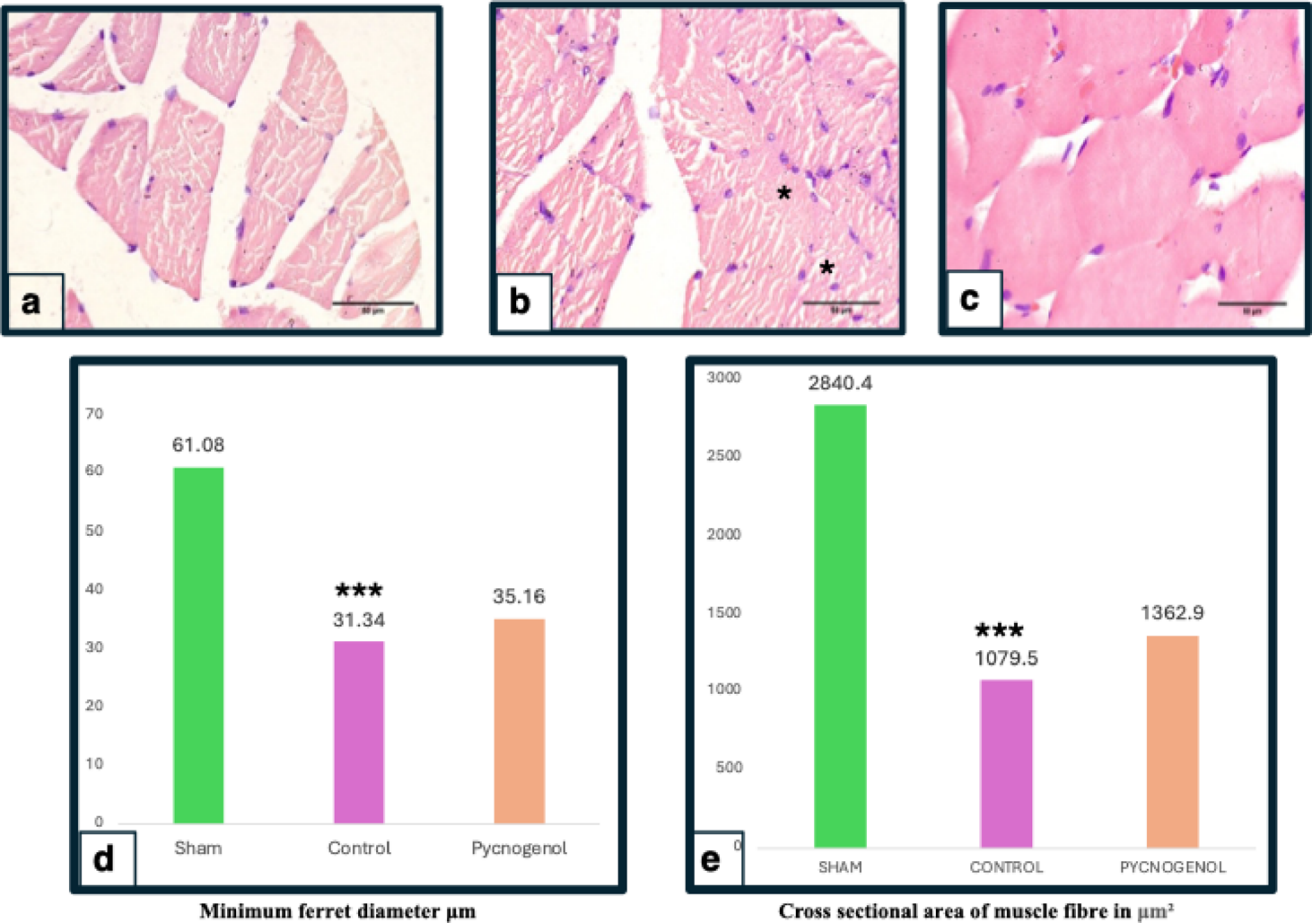
Histological cross-section of gastrocnemius and soleus muscles stained with H&E, viewed under 400x magnification. (**a**) Control group showing reduced fibre area and minimum ferret diameter (1079.5 ± 135.5 μm², 31.34 ± 1.30 μm), (**b**) Pycnogenol group with increased fibre area and minimum ferret diameter compared to control group(1362.9 ± 108.2 μm², 35.16 ± 1.90 μm, ***p < 0.001 vs. Control) and hypertrophy, fibre splitting, and reduced myofiber separation(indicated by *), and (**c**) Sham group with normal morphology (2840.40 ± 127.34 μm²). (**d**) Bar graph showing the distribution of Minimum ferrets diameter (µm) and cross sectional area of muscle fibre (**e**) for Sham, Control, and Pycnogenol groups (*n* = 7 per group). The Pycnogenol group shows statistically significantly higher values compared to Control. Statistical significance assessed using one-way ANOVA with Tukey’s post hoc test (*n* = 7 per group, ***p < 0.001).

Histological analysis (Table 1; Fig. 5) revealed a significant increase in muscle fiber area in the Pycnogenol group (1362.9 ± 108.2 μm²) compared to the Control group (1079.5 ± 135.5 μm², ***p < 0.001). The Minimum Ferret Diameter also increased from 31.34 ± 1.30 μm in the Control group to 35.16 ± 1.90 μm in the Pycnogenol group (***p < 0.001), indicating reduced muscle atrophy.

**Table 1:** Distribution of Sham, Control, and Pycnogenol groups based on muscle and nerve histology.

Variable	Parameters		Study group		*p*-value
		Sham group	Control	Pycnogenol	
Muscle histology	Cross sectional area of muscle fibre(µm²)	2840.40 ± 127.34	1079.5 ± 135.5	1362.9 ± 108.2	< 0.001^***^
	Minimum ferret diameter(µm)	61.08 ± 2.12	31.34 ± 1.30	35.16 ± 1.90	< 0.001^***^
Nerve histology	Number of myelinated nerve fibers.	96.14 ± 2.34	22.85 ± 4.84	47.85 ± 6.49	< 0.001^***^
	Myelination color intensity (greyscale)	231.85 ± 4.20	191.7 ± 5.02	205.57 ± 4.68	< 0.001^***^
	Myelinated fibre density ((number of axons/mm^2^.)	24887.14 ± 978.7	28758.1 ± 778.7	37484.2 ± 1129.2	< 0.001***
	Axon diameter (µm)	4.52 ± 0.12	2.49 ± 0.16	3.18 ± 0.06	< 0.001^***^
	Diameter of myelinated nerve fiber (µm)	8.74 ± 0.43	3.10 ± 026	4.53 ± 0.32	< 0.001^***^
	Myelinated axon area (µm²)	38.63 ± 0.98	20.31 ± 0.85	22.62 ± 3.18	0.088
	Average G ratio	0.54 ± 0.05	0.80 ± 0.065	0.69 ± 0.44	0.004^**^

All values presented as (Mean *±* Standard deviation).*p < 0.05,**p < 0.01,***p < 0.001.

### Nerve histology

**Fig. 6:**
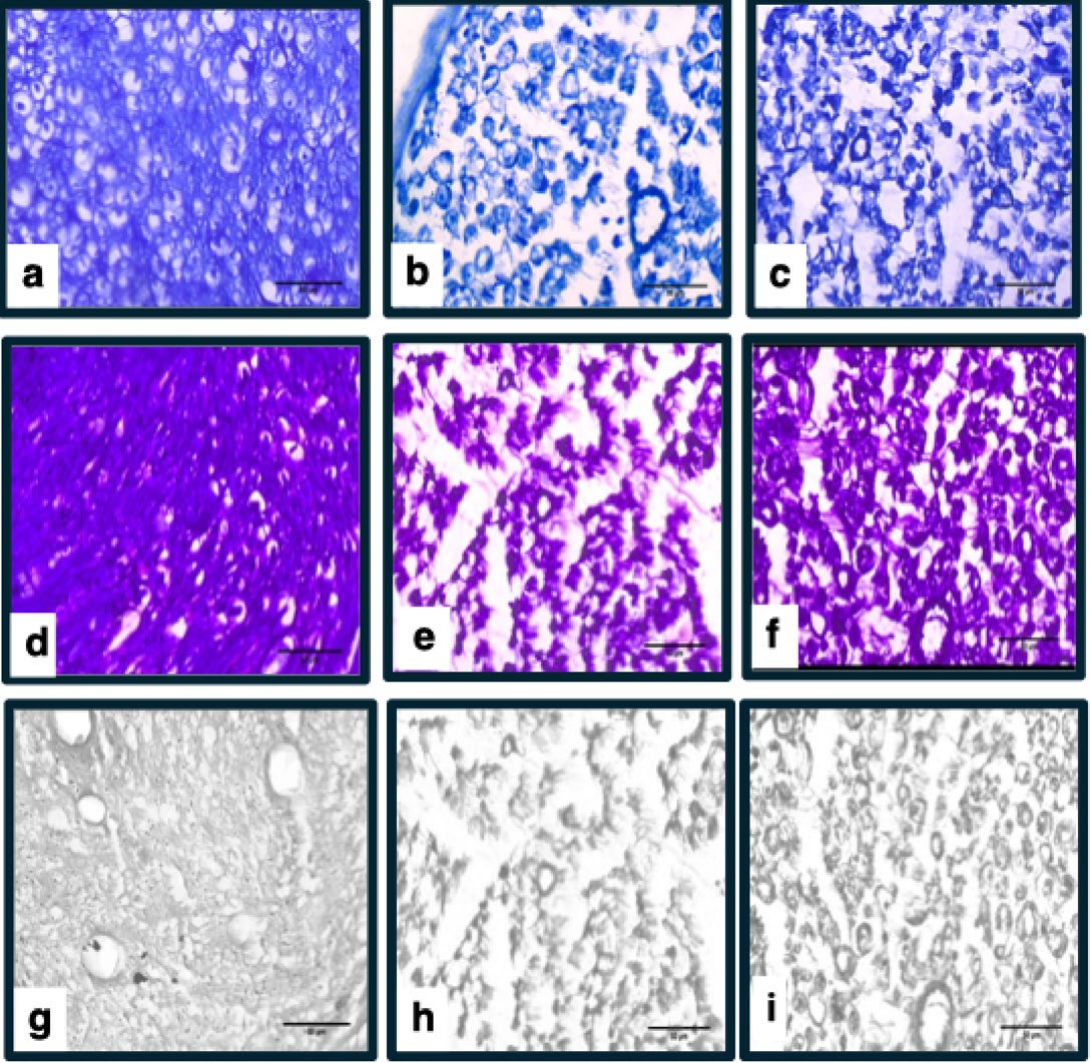
Histological analysis of sciatic nerve by toluidine blue and Luxol fast blue stain viewed under 400x (**a**–**c**) microscopic images of the Transverse section of Toluidine blue stain of the rat Sciatic nerve; Sham, Control and Pycnogenol group respectively. (**d**–**f**) Transverse section of Luxol fast blue stain of the rat Sciatic nerve; Sham, Control and Pycnogenol group respectively. (**g**–**i**) Images from blue channel of image J software; Sham, Control and Pycnogenol groups respectively.

Histological evaluation of sciatic nerve regeneration demonstrated significant improvements in the Pycnogenol-treated group as shown in (Table 1,supplementary Fig. 1). Axon diameter, measured using toluidine blue staining (Fig. 6), was significantly larger in the Pycnogenol group (3.18 ± 0.06 μm) compared to the Control group (2.49 ± 0.16 μm, *p* < 0.001). The number of myelinated nerve fibres, assessed using Luxol fast blue staining(Fig. 6), was also significantly higher in the Pycnogenol group (47.85 ± 6.49) than in the Control group (22.85 ± 4.84, ***p < 0.001) (Tabe 1). Myelination color intensity was also significantly higher in the Pycnogenol group (205.57 ± 4.68) compared to the Control group (191.7 ± 5.02, ***p < 0.001) (Table 1). While the myelinated axon area showed an increase from 20.31 ± 0.85 μm² in the Control group to 22.62 ± 3.18 μm² in the Pycnogenol group, this difference was not statistically significant (*p* = 0.088). However, the diameter of myelinated nerve fibers was significantly greater in the Pycnogenol group (4.53 ± 0.32 μm) than in the Control group (3.10 ± 0.26 μm, ***p < 0.001) (Table 1). Importantly, the average G-ratio, which reflects myelination efficiency, was significantly lower in the Pycnogenol group (0.69 ± 0.44) compared to the Control group (0.80 ± 0.065, **p = 0.004). A lower G-ratio is indicative of improved axonal myelination, reinforcing the beneficial effects of Pycnogenol on nerve regeneration.

## Discussion

This study explored the therapeutic potential of Pycnogenol in promoting sciatic nerve regeneration in inbred female Sprague Dawley rats after a crush injury. Our results demonstrated that Pycnogenol supplementation significantly improved nerve regeneration, myelination, and functional recovery compared to the control group. While previous studies have investigated Pycnogenol’s effects on oxidative stress and inflammation in various tissue injury models^33^, its role in peripheral nerve regeneration remains underexplored. This study provides novel evidence that Pycnogenol not only accelerates functional recovery but also improves structural integrity in injured nerves, making it a promising candidate for peripheral nerve injury therapies. Thus, we discuss the mechanisms underlying these effects comparing with previous research.

Oxidative stress and inflammation are two major pathophysiological factors that hinder nerve regeneration after injury^34^. Pycnogenol is rich in procyanidins, which have been shown to act as free radical scavengers and reduce oxidative damage in injured tissues. Our findings align with the previous studies^35,36^, which demonstrated that Pycnogenol significantly reduces malondialdehyde (MDA) levels, a key biomarker of lipid peroxidation, while increasing total antioxidant capacity (TAS) and total glutathione (tGSH) levels. The observed functional recovery in the Pycnogenol group is consistent with its known anti-inflammatory potential, potentially creating a favourable environment for nerve repair^37,38^. This is supported by findings from^35^ who demonstrated that Pycnogenol reduces neuroinflammation in a cisplatin-induced optic nerve injury model and^35^ who suggested functional recovery of peripheral nerve injury is dependent on the pro-inflammatory cytokines IL-1β and TNF^38^. Thus, by decreasing inflammation and oxidative stress, Pycnogenol may help maintain a pro-regenerative microenvironment essential for axonal recovery.

One of the notable findings of this study was the elevated NGF levels in the Pycnogenol-treated group. NGF is essential for neuronal survival, axonal growth, and Schwann cell activation. The increase in NGF from the proximal nerve terminals observed in our study aligns with previous reports demonstrating its involvement in remyelination and debris clearance via the p75^NTR^/AMPK/mTOR signalling pathway^39^. Schwann cells are known to mediate these processes by clearing myelin debris and secreting neurotrophic factors^40,41^. Although Schwann cells are a major source of NGF following nerve injury, other cell types such as fibroblasts, macrophages, and even injured neurons also contribute to its production^40–42^. While we did not directly assess Schwann cell activity, the elevated NGF levels, along with histological improvements such as lower G-ratio^43^, increased axon diameter, and higher fibre density, may reflect an overall enhancement of the regenerative microenvironment. These findings are consistent with literature highlighting NGF’s multifaceted role in coordinating neurotrophic support, including effects on Schwann cells and other supporting cells involved in peripheral nerve repair^42^.

The Sciatic Functional Index (SFI) scores demonstrated significant motor recovery in Pycnogenol-treated animals. By Day 14, the Pycnogenol group exhibited significantly lower SFI values, indicating better locomotor function than the control group. This trend continued through Day 28, reinforcing the hypothesis that Pycnogenol accelerates functional nerve regeneration. These findings are in line with previous studies on natural antioxidants, which have shown similar functional improvements in models of nerve injury and repair^44,45^. Sensory recovery was also notably improved in the Pycnogenol-treated group, as indicated by the pinprick and cold allodynia tests. The earlier return of sensory responses suggests that Pycnogenol may enhance sensory nerve fibre regeneration, potentially by reducing inflammatory hypersensitivity that can delay functional recovery.

Denervation-induced muscle atrophy is a major concern following peripheral nerve injuries. Our study demonstrated that Pycnogenol significantly preserved muscle mass, as evidenced by the increased wet muscle weight, fibre area, and ferret diameter compared to the control group. These results are consistent with^35^ who reported that Pycnogenol reduces oxidative muscle damage and preserves muscle architecture after optic nerve injury. The apparent ability of Pycnogenol to reduce muscle-wasting may be attributed to its potential role in attenuating oxidative stress and inflammatory responses, which are key contributors to muscle atrophy following nerve injury^46^. The findings complement research by^47^, which demonstrated that preserving muscle integrity contributes to improved functional recovery by facilitating motor neuron reinnervation.

Histological analyses revealed significantly improved axonal regeneration, fibre density, and myelin integrity in the Pycnogenol-treated group. The g-ratio, a measure of myelination efficiency, was significantly lower in the Pycnogenol group, suggesting enhanced myelin formation. These findings align with^41^ who demonstrated that NGF enhances myelin clearance and promotes axonal remyelination via Schwann cells. Moreover, our study demonstrated a significant increase in the diameter of myelinated nerve fibres, suggesting that Pycnogenol may not only support axon regrowth but also facilitates proper myelination, which is essential for efficient nerve conduction and functional recovery. Pycnogenol may promote improved myelination by increasing the expression of growth-associated proteins such as GAP-43(Growth Associated Protein 43), MAG(Myelin-Associated Glycoprotein), synapsin-1, and PSD-95(Postsynaptic Density Protein 95)^39^. These proteins are essential for the stabilization of axons and the enhancement of conduction velocity, further facilitating nerve regeneration and improving overall nerve function. These findings add to the growing body of evidence that bioactive compounds with antioxidant and neurotrophic properties may have clinical applications in nerve injury treatments.

Recent research has emphasized additional therapeutic benefits of Pycnogenol, such as its antifibrotic properties and ability to stabilize mitochondria, which might enhance its neuroprotective capabilities in the regeneration of peripheral nerves^48^. Pycnogenol has shown its antifibrotic properties in a mouse model treated with polyhexamethylene guanidine by influencing fibrotic markers and decreasing excessive extracellular matrix accumulation, vital for avoiding fibrosis-related barriers to nerve regeneration^49^. Fibrotic scarring frequently acts as a physical and biochemical obstacle to axonal regeneration, and Pycnogenol’s capacity to lessen this process indicates a positive influence in fostering a more conducive setting for nerve healing^50^. Additionally, Pycnogenol has been noted to stabilize mitochondrial membrane potential, which is essential for sustaining cellular energy balance and inhibiting apoptosis. Previous studies showed that Pycnogenol has neuroprotective properties in spinal cord injury through the prevention of mitochondrial dysfunction^48^. Since mitochondrial stability is vital for the survival and regeneration of axons, this characteristic may additionally bolster its function in aiding nerve repair. Mitochondrial impairment causes increased oxidative stress and energy shortages, which both obstruct Schwann cell function and axonal regeneration^50^. Overall, the apparent beneficial effects of Pycnogenol in nerve regeneration may be mediated by its potential ability to reduce oxidative stress, enhance NGF-mediated nerve regeneration, modulate the inflammatory response, preserve muscle mass, and promote myelination. These potential combined mechanisms, supported by our demonstrated functional recovery, enhanced myelination, and elevated NGF levels, suggest Pycnogenol represents a promising therapeutic agent for nerve repair and regeneration. The use of multiple outcome measures—including behavioural assessments (SFI, sensory testing), histological analysis (axon diameter, fibre density, G-ratio), and biochemical evaluation (NGF quantification)—strengthens the validity of our findings and provides comprehensive evidence for Pycnogenol’s neuroprotective effects.

## Conclusion

This study provides compelling evidence that Pycnogenol significantly enhances sciatic nerve regeneration, muscle preservation, and functional recovery in a rat model of crush injury. The observed improvements, potentially mediated by its antioxidant and anti-inflammatory properties and increased NGF expression, suggest its potential as a therapeutic agent for peripheral nerve injuries. Pycnogenol’s established safety profile and multifaceted pharmacological benefits suggest it may be a promising candidate for advancing treatment strategies in regenerative medicine and neurology.

### Limitations

While this study provides compelling evidence for Pycnogenol’s neuroprotective effects using comprehensive behavioural, histological, and biochemical assessments, several limitations should be acknowledged. The 28-day follow-up period, though sufficient to demonstrate significant functional improvements, captures only early regenerative phases and does not address long-term recovery or potential late complications. Although we observed robust effects with 100 mg/kg/day dosing, testing only a single dose prevents optimal therapeutic window determination and dose-response relationship evaluation. Our tissue homogenate approach successfully quantified NGF elevation but cannot identify specific cellular sources or spatial distribution patterns that would provide deeper mechanistic insights. The absence of molecular pathway analysis, while not reducing the significance of our functional and structural findings, limits mechanistic understanding of Pycnogenol’s neuroprotective cascade in peripheral nerve regeneration.

### Future directions

Building on these promising findings demonstrating significant functional, histological, and biochemical improvements, future research should expand our understanding through systematic clinical translation. Dose-optimization studies would help establish therapeutic windows that maximize efficacy while maintaining the excellent safety profile observed with Pycnogenol. Long-term follow-up investigations extending 3–6 months would determine whether the substantial early improvements we documented translate into sustained functional recovery. The robust NGF elevation observed warrants mechanistic investigations including immunohistochemistry, cytokine profiling, and molecular pathway analysis to identify specific cellular targets and signalling cascades underlying the beneficial effects. The significant functional improvements observed suggest potential for combination therapies with other neuroprotective agents, stem cell therapies, or rehabilitation protocols that could yield synergistic benefits. Finally, the substantial neuroprotective effects demonstrated in this rodent model provide strong rationale for translational studies progressing through larger animal models to human clinical trials evaluating safety, optimal dosing, and efficacy across different peripheral nerve injury types.

## Methods

### Animals

All experimental protocols for the study were approved by the Institutional Animal Ethics Committee (IAEC) of Kasturba Medical College, Manipal (project proposal no. IAEC/KMC/07/2024). The inbred female Sprague Dawley rats (8–10 weeks old) were obtained from the Central Animal Research House Facility, Manipal Academy of Higher Education, Manipal (IAEC Reg. no. 94/PO/RReBi/S/99/CPCSEA) and housed in a clean, well-ventilated room under a 12-hour light/dark cycle with ad libitum access to food and water. All methods were carried out in accordance with American Veterinary Medical Association (AVMA) and Committee for Purpose of Control and Supervision of Experiments on Animals (CPCSEA), Government of India guidelines. The reporting was done in accordance with the ARRIVE 2.0 guidelines (Animal Research: Reporting of In Vivo Experiments)^51^. At the time of surgery, the rats were weighed to ensure they were within the 200–250 g range. After surgery, the animals were housed individually to prevent potential bite injuries to the denervated limb by cage mates. Behavioural evaluation training was conducted for one week before surgery. The animals were randomly assigned to three groups (*n* = 7 per group), and all three groups were given diet ad libitum.

Sham group (Group 1): Rats did not undergo any surgical intervention. Control group (Group 2): Rats underwent sciatic nerve crush injury but did not receive herbal supplementation. Pycnogenol (Pycno) group (Group 3): Rats underwent sciatic nerve crush injury and received Pycnogenol supplementation (100 mg/kg/day) for 28 days.

Animals were randomly allocated into various groups using the GraphPad QuickCalcs online random number generator by a designated investigator, who also administered treatments based on the randomization schedule. A separate investigator performed the anaesthesia and surgical procedures according to the group assignments. All personnel involved in data collection and analysis were blinded to the treatment groups to ensure unbiased results. Treatment codes were revealed only after statistical analysis completion.

### Pycnogenol supplementation

Pycnogenol was obtained from *Natural Factors French Maritime Pine Bark Extract* (25 mg of powdered extract per capsule), standardized to contain 70 ± 5% procyanidins in compliance with the United States Pharmacopeia (USP). The extract was purchased from Horphag Research (Geneva, Switzerland). Pycnogenol (100 mg/kg body weight) was mixed with distilled water (5 ml) to prepare a homogenized solution. This homogenized Pycnogenol solution was administered via 18-gauge feeding needle inserted 4–5 cm into the oesophagus, starting on post-operative day 1.

The present study aims to determine whether 100 mg/kg/day provides superior neuroprotection in a nerve injury model. The 100 mg/kg/day dose was selected based on established safety data demonstrating no toxicity, carcinogenicity, or teratogenicity at doses up to 5 g/kgbwt according to Organisation for Economic Co-operation and Development (OECD)guidelines[Pycnogenol - American Botanical Council, 2019)^18^. While lower doses (10–50 mg/kg/day) have shown efficacy in diabetic neuropathy models for improving nerve conduction velocity and reducing oxidative stress markers^50^, we selected a higher dose(i, e.100 mg/kg/day to maximize potential neuroprotective effects in acute nerve injury.

### Pre-surgical workup

During the one week preceding the surgery, the rats were trained to traverse a walking track to assess the sciatic functional index (SFI)^52^. The hind paws were pressed onto an inked stamp pad before the rats walked across paper placed on the floor. Baseline SFI measurements were obtained one day before surgery. Sensory assessment (pin prick and cold allodynia) was also conducted before surgery, ensuring that a proper baseline value was achieved. All surgical procedures were performed under aseptic conditions. Anaesthesia was induced via intraperitoneal injection of a cocktail containing xylazine (10 mg/kg body weight) and ketamine (75 mg/kg body weight).

### Induction of sciatic nerve crush injury

The left sciatic nerve was exposed via a muscle-splitting incision between the biceps femoris and semitendinosus muscles and then crushed 3 cm proximal to the division of the sciatic nerve into the tibial and common peroneal nerves. The round haemostatic artery forceps was locked at the final notch, and the sciatic nerve was crushed for 30 s, followed by another 30-second crush at the same site but perpendicular to the initial injury(Supplementary Fig. 2). To maintain uniformity of injury across all animals in each group, the following standardized procedures were followed (1) All surgeries performed by the same investigator, (2) A haemostatic artery forceps was locked at the final notch to produce a consistent crush, (3) Crush duration precisely timed using digital stopwatch, (4) Post-crush nerve appearance photographed for verification, (5) Complete functional deficit confirmed within 24 h by absence of toe spreading response. This technique guarantees a complete nerve crush and the degeneration of all distal axons^22,53^.The nerve was observed to ascertain that the epineurium was intact and that the nerve was completely crushed. The nerve continuity remained intact, but the crushed site appeared flattened as shown in Supplementary Fig. 3. The muscle and skin were sutured in layers using 4 − 0 nylon and the rat was placed on a heating pad to keep it warm. Post-operatively, bupivacaine (0.4–0.8 mL/kg of 0.25%) was infiltrated at the surgical site for analgesia, followed by a subcutaneous injection of gentamycin (2–4 mg/kg). A once-daily dose of carprofen was administered for 5 days. The rats were monitored until they recovered from the anaesthesia. After this, the rats were placed in their respective cages. Each rat was observed postoperatively to confirm complete crushing of the nerve indicated by paralysis of the muscles of the foot and complete absence of toe spreading.

### Sensorimotor assessments

These were performed weekly to evaluate sensorimotor function recovery.

### Motor assessment – Sciatic Functional Index (SFI)^52,54^

SFI was measured on post-injury days 7, 14, 21, and 28 in all groups. Three measurements were recorded from normal (N) and operated (E) limbs:

1. Print length (PL) – Distance from heel to third toe.
2. Intermediate toe spread (ITS) – Distance between the second and fourth toes.
3. Toe spread (TS) – Distance between the first and fifth toes.

SFI was calculated using the formula:

$$\:SFI\:=-38.3\:\text{x}\:\frac{EPL-NPL}{NPL}+109.5\:\text{x}\:\frac{ETS-NTS}{NTS}+13.3\:\text{x}\:\frac{EIT-NIT}{NIT}-8.8\:$$

where an SFI of −100 indicates complete impairment, and 0 represents normal function.

### Sensory assessments

- Skin pin prick test^55^: Conducted postoperatively on days 21 and 28 using a No. 4 Von Frey filament on the plantar surface of the injured paw. The response were graded as:

- (-)- No response.
- (+) Mild response (weak retraction/trembling).
- (++) Moderate response.
- (+++) Normal response (similar to the uninjured side).

- Cold allodynia test^54^: Conducted post operatively on days 21 and 28. A drop of acetone was applied to the plantar surface of the injured hind paw, and the withdrawal response was observed for 60 s after application. Paw-licking/shaking was inferred as evidence of sensory recovery.

### Post-euthanasia analysis (Day 28)

For euthanasia, a combination of ketamine (300–360 mg/kg) and xylazine (30–40 mg/kg) was administered intraperitoneally, in accordance with the AVMA Guidelines for the Euthanasia of Animals (2020 Edition).

### Muscle weight measurement

The gastrocnemius and soleus muscles were harvested^46^. The gastrocnemius was detached from the femoral condyles, and the soleus was separated from the Achilles tendon. The muscles were weighed separately using a precision scale. Muscle atrophy was quantified by comparing the weight loss between the injured and uninjured sides. The right and left muscles after the procedure are as shown in supplementary Fig. 4.

### Histopathological assessment of muscle

Muscle samples were fixed in 10% formalin, embedded in paraffin, and sectioned (5–7 μm). Longitudinal and cross-sections were stained with hematoxylin and eosin (H&E). Minimum ferrets diameter and cross-sectional area were analysed using ImageJ software (NIH, USA, v1.54 K). Histopathological analysis was performed by two blinded investigators with > 95% inter-rater agreement.)

### Nerve harvest, histopathology, and immunological assays

The sciatic nerves were excised, including both the proximal and distal segments relative to the crush site. The proximal 2-mm segment of the transected nerve was used for immunological analysis (i.e., ELISA) to assess NGF levels. Quantification of nerve growth factor (NGF) levels was performed using a commercially available Rat NGF ELISA kit (Elabscience^®^ฏ, Catalog No: E-EL-R0652, Houston, TX, USA), following the manufacturer’s protocol.The specificity was validated by the manufacturer, with no significant cross-reactivity or interference observed. All samples and standards were assayed in duplicates. Absorbance was measured at 450 ± 2 nm using a microplate reader. The coefficient of variation for intra- and inter-assay precision was reported to be < 10%. A 2-mm distal segment of the sciatic nerve was selected for histopathological evaluation. Standard protocols for light microscopy were followed^56^. The harvested nerve tissues were fixed in 10% buffered formalin, rinsed thoroughly with tap water, dehydrated through a graded series of alcohol, cleared with xylene, and embedded in paraffin. Semi-thin Sect. (2 μm) were prepared and stained with toluidine blue for axonal evaluation and Luxol fast blue for assessing myelin integrity. Microscopic examination was carried out at 100× and 400× magnification using a Labomed trinocular research microscope, equipped with a MiaCam CMOS AR 6 Pro camera and Image AR Pro software for image capture and analysis. In each animal, five non-overlapping microscopic fields, each spanning 1 mm², were selected at 400× magnification for analysis. All measurements were performed by two independent, blinded observers. Toluidine blue staining was utilized to quantify axon diameter (µm). Luxol fast blue staining was employed to determine myelinated fibre diameter (axon plus myelin), fiber density, fiber area, and the number of myelinated fibres per 1000 μm². To calculate the G-ratio (a marker of myelination efficiency), inner axonal diameters were measured from toluidine blue-stained sections, while outer diameters of myelinated fibres were obtained from the corresponding Luxol fast blue-stained sections. The G-ratio, defined as the ratio of the inner axon diameter to the total fiber diameter, was used to evaluate the degree of myelination, with lower values indicating enhanced myelination.

Quantitative image analysis was conducted using ImageJ software (NIH, USA, version 1.54k). For Luxol fast blue-stained images, the blue channel was isolated to enhance stain visibility and improve measurement accuracy. The following standardized protocol was applied:

1. Images were converted to 8-bit grayscale,
2. Contrast was enhanced via histogram equalization,
3. Thresholds were set at 50–255 to identify fibres,
4. Particle analysis was conducted with a size filter of 10–1000 μm²,circularity range 0.30–1.00.

All quantifications were performed by two blinded observers, achieving > 95% inter-rater agreement.

**Fig. 7:**
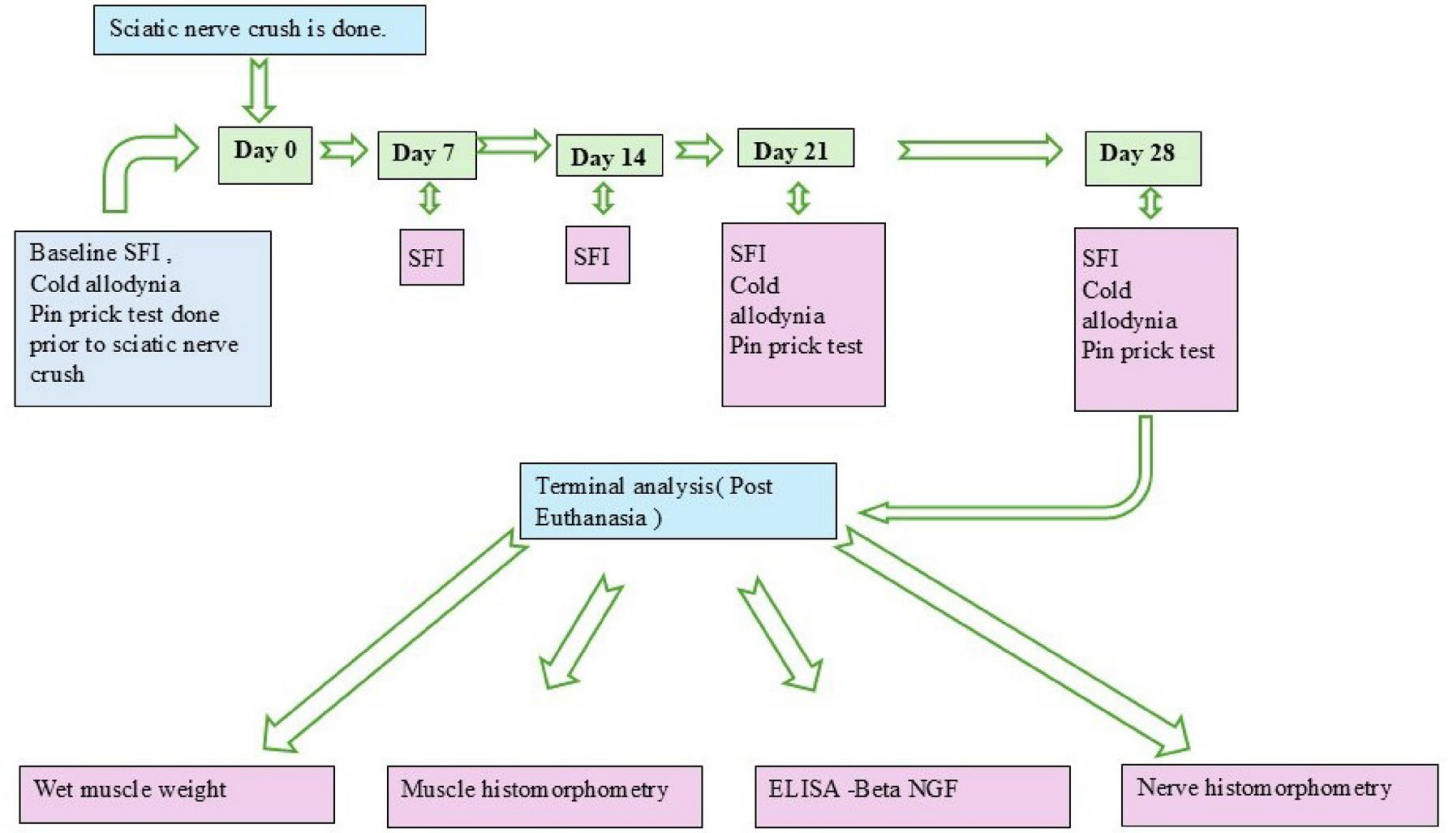
Timeline of the procedures and assessments done on the rats.

A timeline of all procedures and assessments performed on the rats is presented in Fig. 7.

### Statistical analysis

Data analysis was performed using IBM SPSS Statistics version 27.0 (IBM Corp.) and Microsoft Excel for statistical computations and data visualization. All continuous variables were expressed as mean ± standard deviation (SD) to summarize central tendency and variability. To evaluate differences among multiple groups, one-way analysis of variance (ANOVA) was conducted, which allowed for the comparison of means across the different experimental conditions. When a statistically significant difference was detected through ANOVA, Tukey’s post hoc test was applied to perform pairwise comparisons, ensuring that specific group differences were accurately identified while controlling for Type I errors. For comparisons between only two groups, independent t-tests were employed to determine whether there was a statistically significant difference in means. The assumption of normality was assessed before conducting parametric tests to ensure the validity of the results. In all statistical analyses, a p-value < 0.05 was considered the threshold for statistical significance, indicating that results below this value were unlikely to have occurred due to random chance alone. The statistical approach ensured rigorous evaluation of data, minimizing biases, and enhancing the reliability of findings.

## Supplementary Information

Below is the link to the electronic supplementary material.

Supplementary Material 1

## Data Availability

The datasets used and/or analyzed during the current study are available from the corresponding author on reasonable request.

## References

[1] Gaudet, A. D., Popovich, P. G. & Ramer, M. S. Wallerian degeneration: gaining perspective on inflammatory events after peripheral nerve injury. *J. Neuroinflammation* **8**, 110. 10.1186/1742-2094-8-110 (2011). 21878126 10.1186/1742-2094-8-110 PMC3180276

[2] Benga, A., Zor, F., Korkmaz, A., Marinescu, B. & Gorantla, V. The neurochemistry of peripheral nerve regeneration. *Indian J. Plast. Surg. Off Publ Assoc. Plast. Surg. India*. **50**, 5–15 (2017). 10.4103/ijps.IJPS_14_17 PMC5469235 28615804

[3] Carvalho, C. R., Oliveira, J. M. & Reis, R. L. Modern trends for peripheral nerve repair and regeneration: beyond the Hollow nerve guidance conduit. *Front. Bioeng. Biotechnol.* **7**, 337 (2019). 31824934 10.3389/fbioe.2019.00337 PMC6882937

[4] Ciaramitaro, P. et al. Traumatic peripheral nerve injuries: epidemiological findings, neuropathic pain and quality of life in 158 patients. *J. Peripher Nerv. Syst. JPNS*. **15**, 120–127 (2010). 20626775 10.1111/j.1529-8027.2010.00260.x

[5] Yow, Y. Y. et al. Therapeutic potential of complementary and alternative medicines in peripheral nerve regeneration: A systematic review. *Cells* **10**, 2194 (2021). 34571842 10.3390/cells10092194 PMC8472132

[6] Lopes, B. et al. Peripheral nerve injury treatments and advances: one health perspective. *Int. J. Mol. Sci.* **23**, 918 (2022). 35055104 10.3390/ijms23020918 PMC8779751

[7] Ezzat, S. M., Jeevanandam, J., Egbuna, C., Kumar, S. & Ifemeje, J. C. Phytochemicals as sources of drugs. 3–22 10.1007/978-981-13-6920-9_1 (2019).

[8] Choudhari, A. S., Mandave, P. C., Deshpande, M., Ranjekar, P. & Prakash, O. Phytochemicals in cancer treatment: from preclinical studies to clinical practice. *Front. Pharmacol.* **10**, (2020). 10.3389/fphar.2020.00175 PMC7058694 32184731

[9] Swain, S. S., Hussain, T. & Pati, S. Drug-lead Anti-tuberculosis phytochemicals: A systematic review. *Curr. Top. Med. Chem.* **21**, 1832–1868 (2021). 34225624 10.2174/1568026621666210705170510

[10] Caillaud, M. et al. Local low dose Curcumin treatment improves functional recovery and remyelination in a rat model of sciatic nerve crush through Inhibition of oxidative stress. *Neuropharmacol. [Internet]*. **139**, 98–116 (2018). 10.1016/j.neuropharm.2018.07.001 30018000

[11] Chen, M-M. et al. Quercetin promotes motor and sensory function recovery following sciatic nerve-crush injury in C57BL/6J mice. *J. Nutritional Biochem. [Internet]*. **46**, 57–67 (2017). 10.1016/j.jnutbio.2017.04.006 28458138

[12] Sharma, S. & Bhatia, V. Phytochemicals for drug discovery in alzheimer’s disease: in Silico advances. *Curr. Pharm. Des.* **27**, 2848–2860 (2021). 32988343 10.2174/1381612826666200928161721

[13] Liu, Y. et al. The phytochemical potential for brain disease therapy and the possible nanodelivery solutions for brain access. *Front. Oncol.* **12**, 936054 (2022). 35814371 10.3389/fonc.2022.936054 PMC9259986

[14] Li, X. et al. Anti-Parkinson’s disease activity of sanghuangprous vaninii extracts in the MPTP-Induced zebrafish model. *ACS Chem. Neurosci.* **13**, 330–339 (2022). 35044760 10.1021/acschemneuro.1c00656

[15] Souza, N. M. et al. Revisiting the role of biologically active natural and synthetic compounds as an intervention to treat injured nerves. *Mol. Neurobiol.* **58**, 4980–4998 (2021). 34228268 10.1007/s12035-021-02473-z

[16] Rohdewald, P. Pycnogenol^®^ - Scientific File. Section 19 (Horphag Research, 2005).

[17] Li, Y. Y., Feng, J., Zhang, X. L. & Cui, Y. Y. Pine bark extracts: Nutraceutical, Pharmacological, and toxicological evaluation. *J. Pharmacol. Exp. Ther.* **353**, 9–16 (2015). 25597308 10.1124/jpet.114.220277

[18] Organisation for Economic Co-operation and Development (OECD). *Test No. 423: Acute Oral Toxicity – Acute Toxic Class Method. OECD Guidelines for the Testing of Chemicals, Sect. 4. Health Effects* 10.1787/9789264071001-en (OECD Publishing, 2002).

[19] Organisation for Economic Co-operation and Development (OECD). *Test No. 408: Repeated Dose 90-Day Oral Toxicity Study in Rodents. OECD Guidelines for the Testing of Chemicals, Sect. 4. Health Effects *10.1787/9789264070707-en (OECD Publishing, 1998).

[20] Devaraj, S. et al. Supplementation with a pine bark extract rich in polyphenols increases plasma antioxidant capacity and alters the plasma lipoprotein profile. *Lipids* **37**, 931–934 (2002). 12530550 10.1007/s11745-006-0982-3

[21] Canali, R., Comitato, R., Schonlau, F. & Virgili, F. The anti-inflammatory Pharmacology of pycnogenol in humans involves COX-2 and 5-LOX mRNA expression in leukocytes. *Int. Immunopharmacol.* **9**, 1145–1149 (2009). 19508901 10.1016/j.intimp.2009.06.001

[22] Enseleit, F. et al. Effects of pycnogenol on endothelial function in patients with stable coronary artery disease: a double-blind, randomized, placebo-controlled, cross-over study. *Eur. Heart J.* **33**, 1589–1597 (2012). 22240497 10.1093/eurheartj/ehr482

[23] Liu, X. et al. Antidiabetic effect of pycnogenol French maritime pine bark extract in patients with diabetes type II. *Life Sci.* **75**, 2505–2513 (2004). 15363656 10.1016/j.lfs.2003.10.043

[24] Robertson, N. U., Schoonees, A., Brand, A. & Visser, J. Pine bark (Pinus spp.) extract for treating chronic disorders. *Cochrane Database Syst. Rev.* **2020**, CD008294 (2020). 10.1002/14651858.CD008294.pub5 PMC8094515 32990945

[25] Roseff, S. J. Improvement in sperm quality and function with French maritime pine tree bark extract. *J. Reprod. Med.* **47**, 821–824 (2002). 12418064

[26] Luyens, B., Felgueroso-Bueno, F. & Massat, I. Beneficial effects of Pycnogenol^®^ on attention deficit hyperactivity disorder (ADHD): A review of clinical outcomes and mechanistic insights. *Arch. Pediatr.* **9**, 317. 10.29011/2575-825X.100317 (2024).

[27] Paarmann, K. et al. French maritime pine bark treatment decelerates plaque development and improves Spatial memory in alzheimer’s disease mice. *Phytomedicine* **57**, 39–48. 10.1016/j.phymed.2018.11.033 (2019). 30668321 10.1016/j.phymed.2018.11.033

[28] Norris, C. M., Sompol, P., Roberts, K. N., Ansari, M. & Scheff, S. W. Pycnogenol protects CA3-CA1 synaptic function in a rat model of traumatic brain injury. *Exp. Neurol.* **276**, 5–12. 10.1016/j.expneurol.2015.11.006 (2016). 26607913 10.1016/j.expneurol.2015.11.006 PMC4715929

[29] Malekahmadi, M. et al. The effect of French maritime pine bark extract supplementation on inflammation, nutritional and clinical status in critically ill patients with traumatic brain injury: A randomized controlled trial. *Phytother Res. PTR*. **35**, 5178–5188 (2021). 34382717 10.1002/ptr.7187

[30] Martinez, P. V. Neuroprotection by immunomodulatory agents in animal models of Parkinson’s disease. *Neural Regener. Res.* **13**(9), 1493–1506. 10.4103/1673-5374.237108 (2018). 10.4103/1673-5374.237108 PMC6126123 30127102

[31] 26.Belcaro, G. et al. Pycnogenol^®^ supplementation alleviates symptoms of parkinson’s disease with mild cognitive impairment. *J. Neurosurg. Sci.* **66** (4), 371–377. 10.23736/S0390-5616.22.05715-0 (2022). 36153882 10.23736/S0390-5616.22.05715-0

[32] Simpson, T., Kure, C. & Stough, C. Assessing the efficacy and mechanisms of Pycnogenol^®^ on cognitive aging from in vitro animal and human studies. *Front. Pharmacol.* **10**, 694. 10.3389/fphar.2019.00694 (2019). 31333448 10.3389/fphar.2019.00694 PMC6619435

[33] Dash, U. C. et al. Oxidative stress and inflammation in the pathogenesis of neurological disorders: mechanisms and implications. *Acta Pharm. Sin B*. **15**, 15–34 (2025). 40041912 10.1016/j.apsb.2024.10.004 PMC11873663

[34] Erel, O. A novel automated method to measure total antioxidant response against potent free radical reactions. *Clin. Biochem.* **37**, 112–119 (2004). 14725941 10.1016/j.clinbiochem.2003.10.014

[35] Icel, E. et al. Effects of pycnogenol on cisplatin-induced optic nerve injury: an experimental study. *Cutan. Ocul Toxicol.* **37**, 396–400 (2018). 29969298 10.1080/15569527.2018.1495224

[36] Xia, R., Ji, C. & Zhang, L. Neuroprotective effects of pycnogenol against Oxygen-Glucose Deprivation/Reoxygenation-Induced injury in primary rat astrocytes via NF-κB and ERK1/2 MAPK pathways. *Cell. Physiol. Biochem.* **42**, 987–998 (2017). 28662519 10.1159/000478681

[37] Nattagh-Eshtivani, E. et al. The role of pycnogenol in the control of inflammation and oxidative stress in chronic diseases: molecular aspects. *Phytother Res.* **36**, 2352–2374 (2022). 35583807 10.1002/ptr.7454

[38] Nadeau, S. et al. Functional recovery after peripheral nerve injury is dependent on the pro-inflammatory cytokines IL-1β and TNF: implications for neuropathic pain. *J. Neurosci. Off J. Soc. Neurosci.* **31**, 12533–12542 (2011). 10.1523/JNEUROSCI.2840-11.2011 PMC6703268 21880915

[39] Gordon, T. & Nerve Regeneration Understanding biology and its influence on return of function after nerve transfers. *Hand Clin.* **32**, 103–117 (2016). 27094884 10.1016/j.hcl.2015.12.001

[40] Li, R. et al. Nerve growth factor activates autophagy in Schwann cells to enhance Myelin debris clearance and to expedite nerve regeneration. *Theranostics* **10**, 1649–1677 (2020). 32042328 10.7150/thno.40919 PMC6993217

[41] Wei, C. et al. Advances of Schwann cells in peripheral nerve regeneration: from mechanism to cell therapy. *Biomed. Pharmacother*. **175**, 116645 (2024). 38729050 10.1016/j.biopha.2024.116645

[42] Minnone, G., De Benedetti, F. & Bracci-Laudiero, L. NGF and its receptors in the regulation of inflammatory response. *Int. J. Mol. Sci.* **18** (5), 1028. 10.3390/ijms18051028 (2017). Published 2017 May 11. 28492466 10.3390/ijms18051028 PMC5454940

[43] Chomiak, T. & Hu, B. What Is the Optimal Value of the g-Ratio for Myelinated Fibers in the Rat CNS? A Theoretical Approach. *PLoS ONE* **4**, e7754 (2009). 19915661 10.1371/journal.pone.0007754 PMC2771903

[44] Chen, J., Yang, R., Li, H. & Lao, J. Green tea polyphenols promote functional recovery from peripheral nerve injury in rats. *Med. Sci. Monit. Int. Med. J. Exp. Clin. Res.* **26**, e923806 (2020). 10.12659/MSM.923806 PMC7476353 32851993

[45] Scheff, S. W., Ansari, M. A. & Roberts, K. N. Neuroprotective effect of Pycnogenol^®^ following traumatic brain injury. *Exp. Neurol.* **239**, 183–191 (2013). 23059456 10.1016/j.expneurol.2012.09.019 PMC3534537

[46] Yadav, A. & Dabur, R. Skeletal muscle atrophy after sciatic nerve damage: mechanistic insights. *Eur. J. Pharmacol.* **970**, 176506 (2024). 38492879 10.1016/j.ejphar.2024.176506

[47] Gugliandolo, E. et al. Effect of PEA-OXA on neuropathic pain and functional recovery after sciatic nerve crush. *J. Neuroinflammation*. **15**, 264 (2018). 30217164 10.1186/s12974-018-1303-5 PMC6137737

[48] Lin, Y. et al. Pycnogenol protects against spinal cord injury by preventing mitochondrial dysfunction. *Neurosci. Lett.* **715**, 134645. 10.1016/j.neulet.2020.134645 (2020).31765728

[49] Park, J. et al. Antifibrotic effects of pycnogenol in a mouse model treated with polyhexamethylene guanidine. *J. Mol. Med. (Berl)*. **100** (4), 567–578. 10.1007/s00109-022-02134-5 (2022).

[50] Jankyova, S. et al. Pycnogenol^®^ efficiency on glycaemia, motor nerve conduction velocity, and markers of oxidative stress in mild type diabetes in rats. *Phytother Res.* **23** (8), 1169–1174. 10.1002/ptr.2776 (2009). 19165752 10.1002/ptr.2776

[51] Percie du Sert. The ARRIVE guidelines 2.0: updated guidelines for reporting animal research. *PLoS Biol.* **18**, e3000410 (2020). 32663219 10.1371/journal.pbio.3000410 PMC7360023

[52] de Medinaceli, L., Freed, W. J. & Wyatt, R. J. An index of the functional condition of rat sciatic nerve based on measurements made from walking tracks. *Exp. Neurol.* **77** (3), 634–643 (1982). 7117467 10.1016/0014-4886(82)90234-5

[53] Tos, P. et al. Methods and protocols in peripheral nerve regeneration experimental research: part I—experimental models. *Int. Rev. Neurobiol.* **87**, 47–79. 10.1016/S0074-7742(09)87004-9 (2009). 19682633 10.1016/S0074-7742(09)87004-9

[54] Acharya, N. et al. The outcome of polyethylene glycol fusion augmented by electrical stimulation in a delayed setting of nerve repair following neurotmesis in a rat model. *Acta Neurochir. (Wien)*. **165** (12), 3993–4002 (2023). 37907766 10.1007/s00701-023-05854-6 PMC10739326

[55] Paskal, A. M. et al. Neuroregenerative effects of polyethylene glycol and FK-506 in a rat model of sciatic nerve injury. *J. Plast. Reconstr. Aesthet. Surg.* **73** (2), 222–230. 10.1016/j.bjps.2019.10.011 (2020). 31759923 10.1016/j.bjps.2019.10.011

[56] Woodhoo, A. (ed) *Myelin: Methods and Protocols. Methods in Molecular Biology*. 1791 (Springer Science + Business cMedia, part of Springer Nature, 2018).

