# Supplementary material for: Neuroprotective effects of Pycnogenol on nerve regeneration and functional recovery after sciatic nerve crush injury in rodents
Source: Sci Rep. 2025 Oct 30;15:38078. doi: 10.1038/s41598-025-21975-7 (PMC12575757; doi:10.1038/s41598-025-21975-7)
Supplement: Supplementary file 1 — Supplementary Material 1 [file 41598_2025_21975_MOESM1_ESM.docx]

**Supplementary Table 1: Comparison of Sciatic Function Index (SFI) Among the Three Study Groups**

| Time Points | Study Group | | | p-value |
| --- | --- | --- | --- | --- |
|  | **Sham** | **Control** | **Pycnogenol** |  |
| Baseline | 5.01±1.23 | 5.08±2.33 | 5.20±2.78 | 0.935 |
| Day 7 | 4.78±-1.65 | 95.45±2.47 | 96.74±2.24 | 0.325 |
| Day 14 | 3.08±-1.65 | 89.81±2.42 | 83.60±2.26 | <0.001^***^ |
| Day 21 | 2.78±-2.36 | 72.30±2.57 | 64.99±2.30 | <0.001^***^ |
| Day 28 | 2.45±-1.87 | 62.95±2.93 | 49.42±3.0 | <0.001^***^ |

All values presented as (Mean + Standard Deviation).*p<0.05,**p<0.01,***p<0.001

**Supplementary Table 2: Distribution of Response to Cold Allodynia Using Acetone Across Sham, Control, and Pycnogenol Groups at Different Time Points**

| Time Points | Study Group | | | p-value |
| --- | --- | --- | --- | --- |
|  | **Sham** | **Control** | **Pycnogenol** |  |
| Baseline | 55.57±4.32 | 54.85±3.13 | 57.0±2.16 | 0.935 |
| Day 21 | 56.71±3.67 | 12.57±1.71 | 18.85±1.86 | <0.001^***^ |
| Day 28 | 53.76±3.89 | 19.85±1.06 | 22.85±0.89 | <0.001^***^ |

All values presented as (Mean + Standard Deviation).*p<0.05,**p<0.01,***p<0.001

**Supplementary Table 3: Distribution of Sham, Control, and Pycnogenol Groups Based on Wet Muscle Weight:**

| Variable | Parameters |  | Study Group | | p-value |
| --- | --- | --- | --- | --- | --- |
|  |  | **Sham Group** | **Control** | **Pycnogenol** |  |
| Wet muscle weight | Flexor Ratio | 0.95±0.01 | 0.41±0.021 | 0.46±0.03 | 0.006^**^ |
|  | Extensor Ratio | 0.960±0.03 | 0.42±0.01 | 0.47±0.03 | 0.008^**^ |

All values presented as (Mean + Standard Deviation). ).*p<0.05,**p<0.01,***p<0.001

**Supplementary table 4: Distribution of Sham, Control, and Pycnogenol Groups Based on ELISA Beta-NGF Levels:**

| Variable | Parameters |  | Study Group | | p-value |
| --- | --- | --- | --- | --- | --- |
|  |  | **Sham Group** | **Control** | **Pycnogenol** |  |
| Elisa | Beta-NGF (pg/mg) | 1.76±0.21 | 2.05±0.15 | 2.56±0.43 | 0.013^*^ |

**Supplementary Figure 1: (a)***Mean Number of Myelinated Nerve Fiber (Number of axons/mm^2^), (b)Mean Myelination Color Intensity(greyscale), (c)Mean Myelinated Axon Area( μm²) ,(d) Mean Axon Diameter (μm), (e)Diameter of Myelinated Nerve Fiber (μm) ,(f)Mean Myelinated Fibre Density (Number of axons/mm^2^ ) ,(g)Average G Ratio.*

*Its seen that Pycnogenol group showed increase in the number of Myelinated Nerve Fibre, Myelination Color Intensity, Myelinated Fibre Density (μm²), Axon Diameter(μm) and Diameter of Myelinated Nerve Fiber(μm) compared to Control group. Average G Ratio was lower in Pycnogenol group signifying improved nerve conduction compared to Control group. Statistical significance assessed using one-way ANOVA with Tukey’s post hoc test (n=7 per group, *p<0.05,*p<0.01,*p<0.001).*


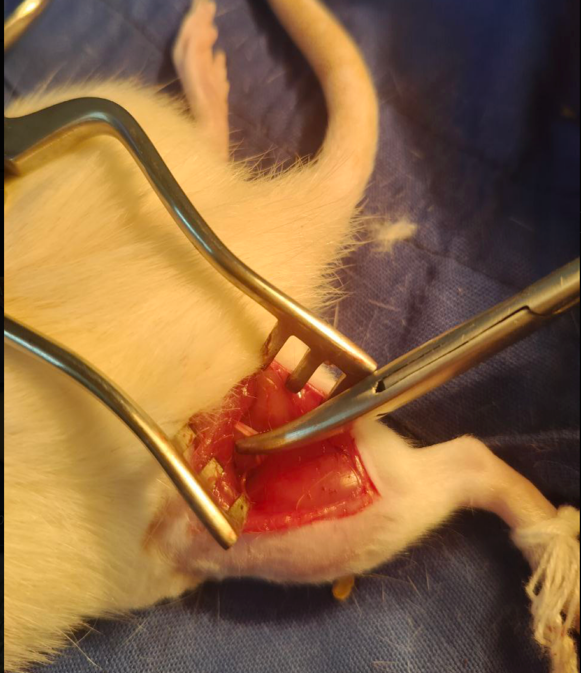


**Supplementary Figure 2:** *Standardized sciatic nerve crush using a round hemostatic forceps for 60 seconds, 3 cm proximal to nerve bifurcation (n=7 per group).*


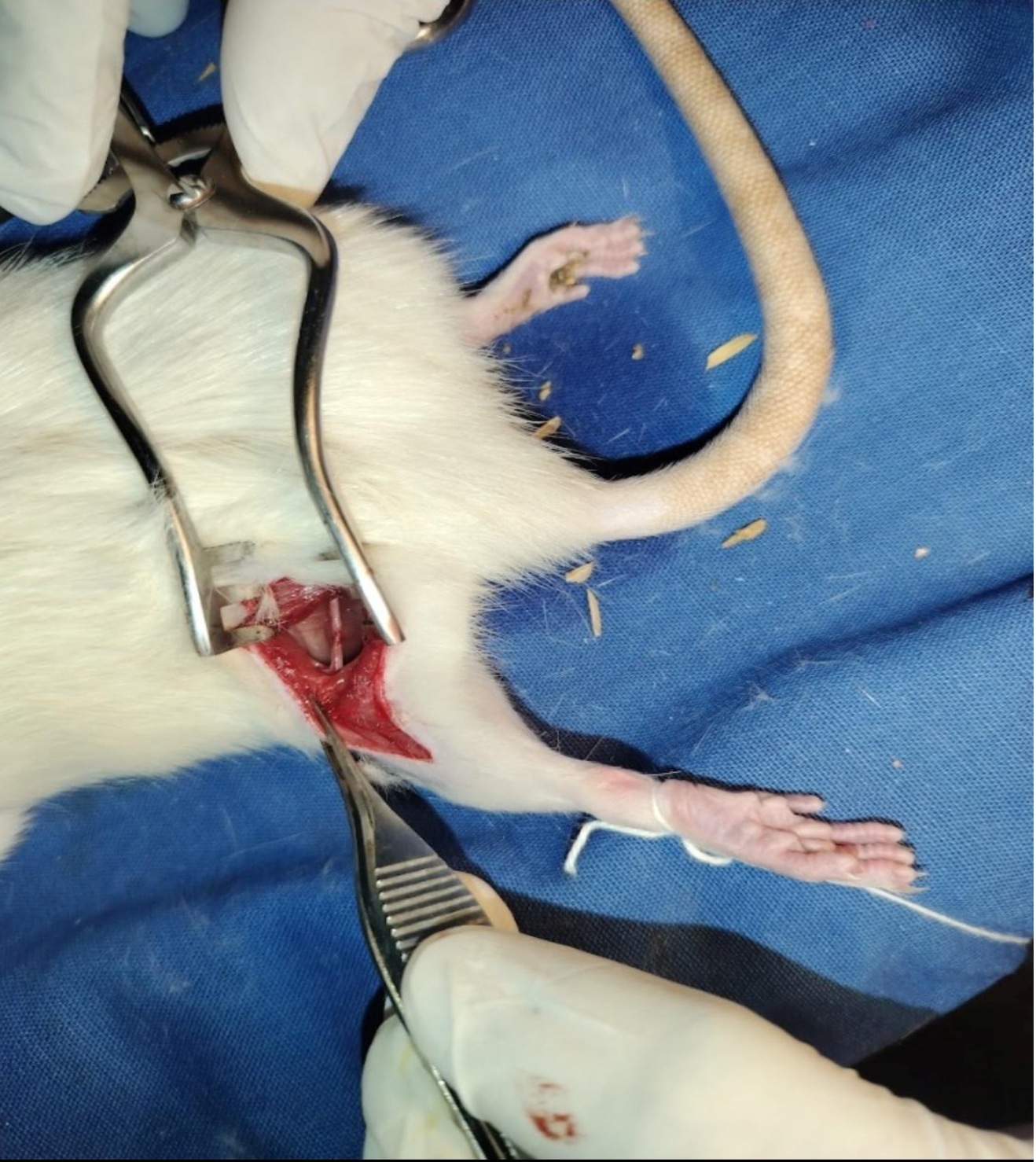


**Supplementary figure 3: Representation of Crush Injury Induced in the Rat Sciatic Nerve**

*Appearance of sciatic nerve after the injury, demonstrating the extent of nerve compression and the affected anatomical region.*


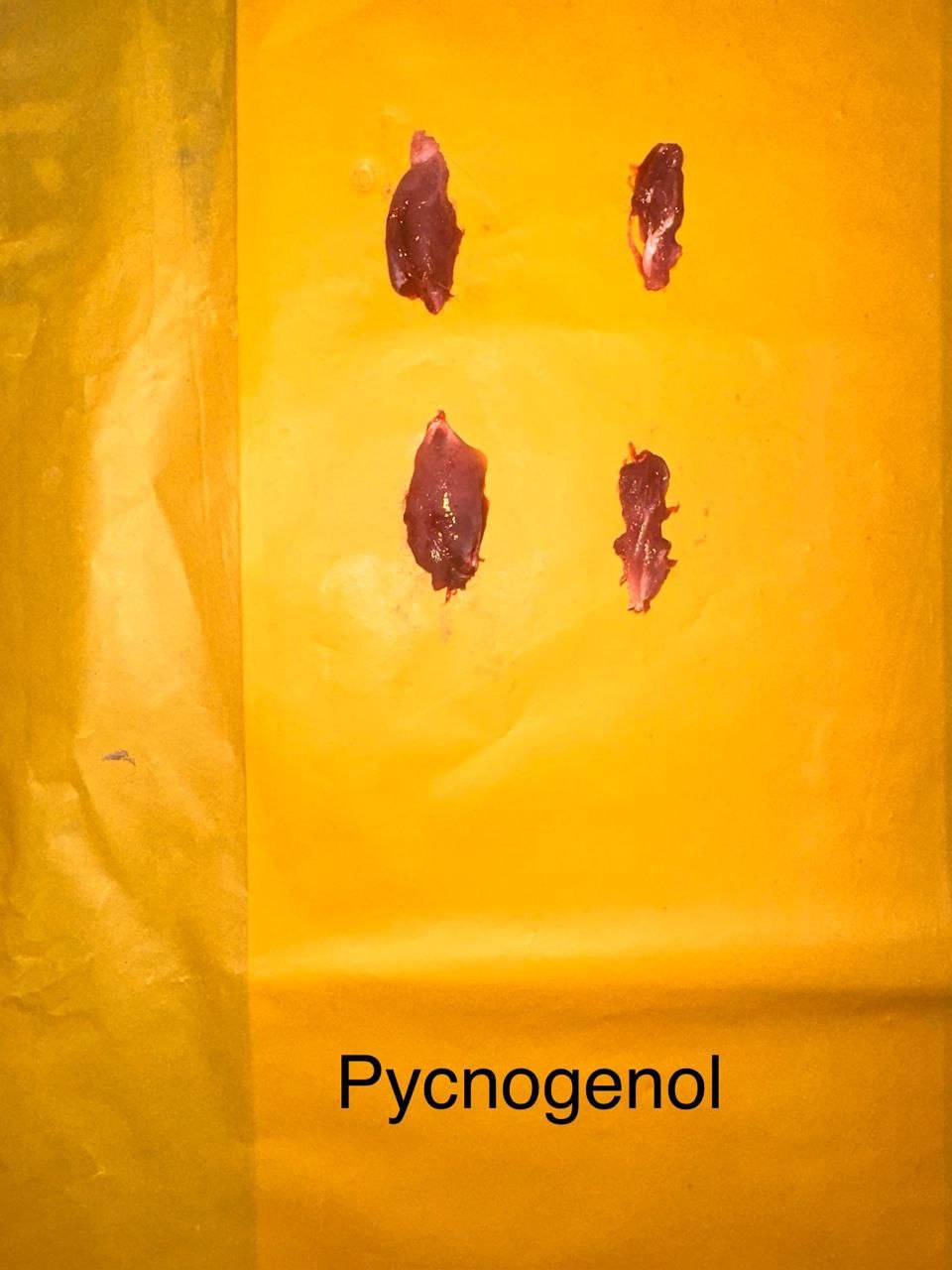


**Supplementary figure 4**:*Wet muscle weight of extensors in the Pycnogenol group comparing the uninjured (left) and injured (right) legs.*
